# Usability and User Experience of an mHealth App for Therapy Support of Patients With Breast Cancer: Mixed Methods Study Using Eye Tracking

**DOI:** 10.2196/50926

**Published:** 2024-03-05

**Authors:** Carolin Anders, Preetha Moorthy, Laura Svensson, Julia Müller, Oliver Heinze, Petra Knaup, Markus Wallwiener, Thomas M Deutsch, Thao-Vy Le, Lina Weinert

**Affiliations:** 1 Institute of Medical Informatics Heidelberg University Hospital Heidelberg Germany; 2 Department of Biomedical Informatics at the Center for Preventive Medicine and Digital Health (CPD) Medical Faculty Mannheim, Heidelberg University Mannheim Germany; 3 Department of Obstetrics and Gynecology Heidelberg University Hospital Heidelberg Germany; 4 Section for Oral Health, Heidelberg Institute of Global Health Heidelberg University Hospital Heidelberg Germany

**Keywords:** mobile health, mHealth, usability, breast cancer, eye tracking, user interface, mixed methods, mobile phone

## Abstract

**Background:**

Early identification of quality of life (QoL) loss and side effects is a key challenge in breast cancer therapy. Digital tools can be helpful components of therapeutic support. *Enable*, a smartphone app, was used in a multicenter, prospective randomized controlled trial in 3 breast cancer centers. The app simultaneously serves as a therapy companion (eg, by displaying appointments), a tool for documenting QoL (eg, by enabling data collection for QoL questionnaires), and documentation of patient-reported side effects. The need for digital tools is continually rising. However, evidence of the effects of long-term use of mobile health (mHealth) apps in aftercare for patients with breast cancer is limited. Therefore, evaluating the usability and understanding the user experience of this mHealth app could potentially contribute valuable insights in this field.

**Objective:**

A usability study was conducted to explore how patients with breast cancer receiving neoadjuvant, adjuvant, or palliative outpatient treatment rated their engagement with the app , the user experience, and the benefits of using the app.

**Methods:**

A mixed methods approach was chosen to combine subjective and objective measures, including an eye-tracking procedure, a standardized usability questionnaire (mHealth App Usability Questionnaire), and semistructured interviews. Participants were surveyed twice during the study period. Interviews were transcribed verbatim and analyzed using thematic analysis. Analysis of the eye-tracking data was carried out using the tracker-integrated software. Descriptive analysis was conducted for the quantitative data.

**Results:**

The mHealth App Usability Questionnaire results (n=105) indicated good overall usability for 2 different time points (4 wk: mean 89.15, SD 9.65; 20 wk: mean 85.57, SD 12.88). The qualitative analysis of the eye-tracking recordings (n=10) and interviews (n=16) showed that users found the *Enable* app easy to use. The design of the app, information about therapies and side effects, and usefulness of the app as a therapy companion were rated positively. Additionally, participants contributed requests for additional app features and suggestions for improving the content and usability of the app. Relevant themes included optimization of the appointment feature, updating the app’s content regularly, and self-administration. In contrast to the app’s current passive method of operation, participants expressed a desire for more active engagement through messaging, alarms, or emails.

**Conclusions:**

The results of this study demonstrate the good usability of the *Enable* app as well as the potential for further development. We concluded from patients’ feedback and requests that mHealth apps could benefit from giving patients a more active role (eg, being able to actively document side effects as they occur). Additionally, regular updates of app content could further contribute to encouraging continued use of mHealth apps. Our findings may also assist other researchers in tailoring their mHealth apps to the actual needs of patients undergoing breast cancer therapy.

## Introduction

### Background

Breast cancer is the most common type of cancer detected in women in the Western world. One in 8 women will develop breast cancer during her lifetime. In Germany, there are 69,000 new cases per year [1]. The diagnosis is a drastic event in the lives of those affected. Although the mortality rate has decreased in recent years, processing and dealing with the new life situation is a great challenge for patients and their social environment [2]. At the onset of therapy, patients can have a strong desire for education and information. Therefore, providing patients with reliable sources of information and support services is a major and important task for the treatment team. Digitalization in medicine offers great potential for supporting the exchange of information and communication between patients and health care providers [3-5]. These benefits can be realized through the use of mobile health (mHealth) apps, which can encompass several helpful functions for patients, such as the provision of educational materials, appointment or medication reminders, and diaries. For the cohort of patients with breast cancer, many of these mHealth apps are already available or are in development [6]. This cohort also shows a high readiness for using health technology, indicating that mHealth apps are an appropriate means of support in the early phase of breast cancer treatment.

**Figure 1:**
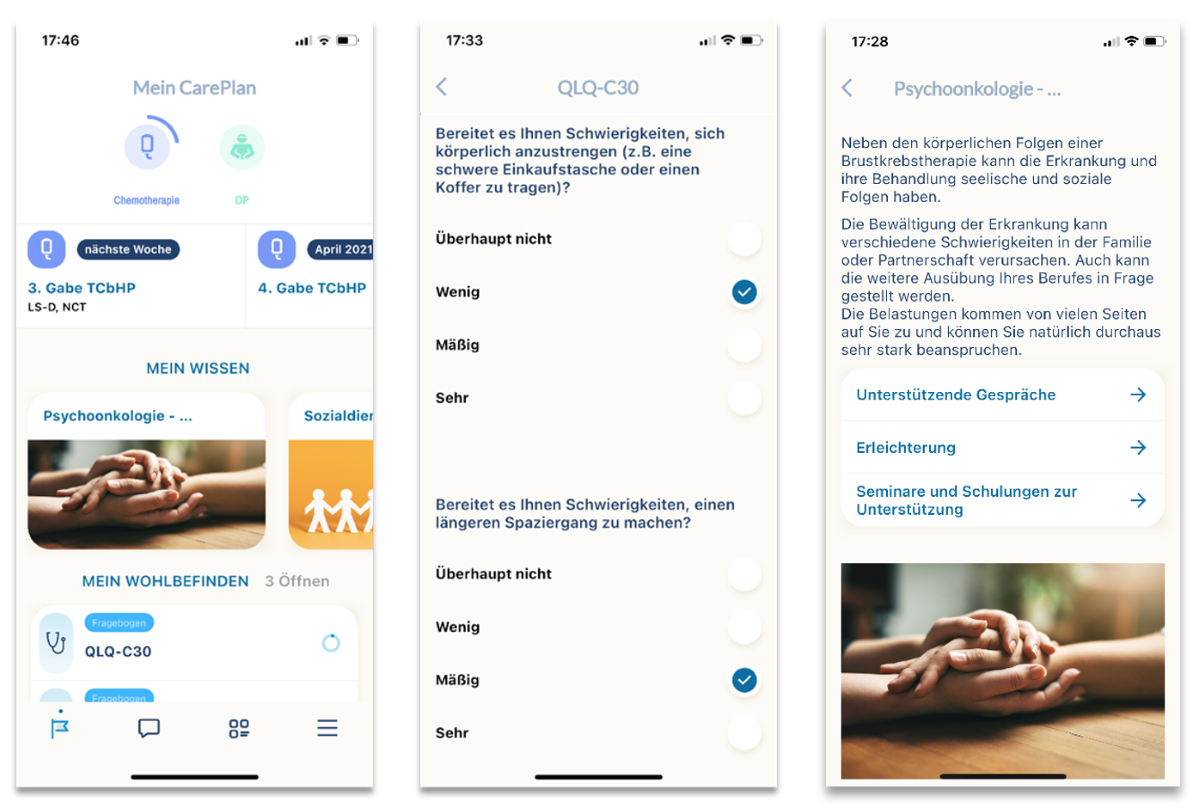
Exemplary content of the Enable app. Left: On the start page of the app, patients can see their current therapy status, a selection of articles from the "My Knowledge" collection, and upcoming questionnaires; Middle: View of the QLQ-C30 questionnaire to assess the quality of life of cancer patients; Right: Sample article on psycho-oncology with the aim of patient education.

A recent study by Chen et al [7] also found that remote monitoring of symptoms between clinical visits could not only improve patient-provider communication but also prepare patients for subsequent chemotherapy cycles and support symptom management. Within the joint *Center for Innovative Care* project, a network of 5 university hospitals in southwest Germany, a new mHealth app for patients with breast cancer was developed. This therapy support tool, called the *Enable* app, aims to combine known benefits of mHealth tools with an innovative reactive assessment of patient-reported outcomes (PROs). It was conceptualized as an iOS or Android mobile app for smartphones and developed by members of the research team with the support of software developers. It includes educational content, information about the side effects of therapies and medications, and information about other support services such as psycho-oncology or nutritional counseling in the form of static text and images. A progress bar illustrates the patient’s individual therapy status in terms of clinical treatment over time (ie, cycles of treatment). In addition to its role as a therapy companion, the app serves as a measurement tool to systematically record patient satisfaction, health-related quality of life (QoL), and patient-reported adverse events. It monitors the neoadjuvant, adjuvant, and follow-up situations in patients with indications for surgery, chemotherapy, radiation, or systemic therapy with primary or metastatic breast cancer. Figure 1 shows exemplary screenshots of the *Enable* app’s start page, the questionnaire display, and information about treatments. As studies have shown that physicians generally underestimate a large proportion of relevant side effects, patients are empowered to report PRO data and side effects directly through the app. In cases of significant treatment-related deterioration, the care team is alerted, and recommendations are sent to the patient. This more relevant treatment information, in turn, helps improve therapy monitoring, treatment quality, and patient satisfaction [8,9].

The clinical outcomes of the use of the *Enable* app were studied in the *ENABLE* randomized controlled trial (RCT). Other research questions addressed in the *ENABLE* RCT related to improving patients’ adherence to therapy, recognizing and treating critical side effects in a timely manner, and measuring the health-related QoL of different therapy strategies. All study participants underwent QoL assessments at 6 time points during and after adjuvant or neoadjuvant chemotherapy. In the intervention group, an additional short weekly EuroQol Visual Analogue Scale questionnaire was administered. In case of deteriorating results, further screening for side effects was triggered, alerting study staff and enabling immediate contact with the patient to provide support in all phases of breast cancer therapy (reactive PRO assessment). The control group received only the app without the reactive PRO assessment.

The body of scientific literature shows that good usability is an important factor for the success of an mHealth app. More specifically, usability can influence patients’ acceptance and adoption of mHealth [10,11]. Usability is defined by Nielsen [12] as a “quality attribute that assesses how easy interfaces are to use.” According to the International Organization for Standardization (ISO) 9241-1, usability is the “extent to which a system, product, or service can be used by specified users to achieve specified goals with effectiveness, efficiency, and satisfaction in a specified context of use” [13]. Although usability focuses exclusively on the process of using an app or device, user experience involves the users’ subjective feelings that result from the use or anticipated use of a system or a product. For the evaluation of mHealth, both concepts are relevant to obtain a comprehensive view of influencing factors [14].

Good usability can help ensure that the app can be used intuitively by patients and health care providers, which in turn improves compliance and increases the effectiveness of the app. A review by Zapata et al [10] demonstrated the importance of adapting mHealth apps to patients’ needs. Relevant usability themes of similar apps were, for example, streamlining of the navigation paths, a clearer information architecture, or the desire for personalization [15,16]. Recent research has also shown that usability assessment is an essential step in the mHealth app development process [17,18]. It is important to ensure that the app is easy to use for the target group and provides the desired benefits [12]. However, a systematic review by Jongerius et al [6] showed that only 1 of 29 mHealth apps for breast cancer care that were studied in their work underwent and published a usability assessment. To address the aforementioned requirements and achieve sustainable and effective use of the *Enable* app, the investigation of usability and user experience is indispensable. Therefore, the study presented in this paper intended to gain an understanding of how patients use the app. The aim was to investigate how patients evaluate their engagement with the app, the user experience, and the benefits of using the app. These findings will serve as a basis for further optimization and adaptation of the app to the patients’ needs.

A mixed methods approach provides the opportunity to collect, triangulate, and analyze qualitative and quantitative data, allowing for the possibility of interpreting the findings from one research approach (ie, qualitative and quantitative) to explain the data generated from the other research approaches. Furthermore, it allows for the use of a qualitative approach to illustrate quantitative findings or the integration of various research approaches to provide a thorough and comprehensive picture of the study [19,20]. Previous studies [15,21,22] have indicated that interviews and usability questionnaires are prevailing methods used for assessing the usability of mHealth apps. However, there are limited studies regarding the real-time capture of users’ visual interactions and the subsequent retrospective analysis of user engagement with mHealth apps through techniques such as eye tracking. Eye tracking, a sensor technology, is used to ascertain an individual’s presence and record their real-time eye movements. This approach is also used to assess the usability of technologies by showcasing decision-making processes through the analysis of eye movement patterns [23,24].

### Objectives

Developing new mHealth apps can be time-consuming and requires several iterations of testing and evaluation. The *ENABLE* project aims to evaluate both the usability and clinical outcomes of the *Enable* app within the same RCT, which could be a promising approach to speed up development, testing, and planning for further implementation. This paper presents a usability study nested within the *ENABLE* RCT and following a mixed methods approach incorporating the eye-tracking method. The objective of this usability study was to explore how patients with breast cancer receiving neoadjuvant, adjuvant, or palliative outpatient treatment rated their engagement with the app, the user experience, and the benefits of using the app.

## Methods

### Study Design

This study was designed following a mixed methods approach combining real-world user experience and standardized observations in a laboratory setting. The study took place at the Department of Obstetrics and Gynecology, Heidelberg University Hospital, Germany.

### Procedure

#### Study Population and Recruitment

The study participants were recruited from the intervention and control groups of the *ENABLE* RCT patient cohort (German Clinical Trials Register—DRKS ID: DRKS00025611). The *ENABLE* RCT had the following inclusion criteria: diagnosis of invasive or metastatic breast cancer and planning of neoadjuvant, adjuvant, or palliative therapy in an outpatient treatment setting (indications for surgery or chemo-, radio-, or systemic therapy); minimum age of 18 years; German language skills; and possession of a smartphone with internet access. Owing to technical requirements for eye tracking, patients wearing bifocals were excluded from participation. At study enrollment, patients were asked about their interest in participating in the usability study. All interested patients at the Department of Obstetrics and Gynecology, Heidelberg University Hospital, Germany, received written and verbal information regarding the content and aim of the study and the respective data protection regulations. On the informed consent form, patients could indicate whether they were interested in participating in the usability aspect of the *ENABLE* RCT. Patients who consented to participate in the nested usability study were contacted individually to schedule appointments for participation following a convenience sampling strategy. No reimbursement was provided. The target sample size was 100 questionnaires, 15 qualitative interviews, and 10 eye-tracking studies. Patient recruitment took place from March 2021 to September 2023.

#### Instruments

The German translation of the mHealth App Usability Questionnaire (MAUQ) [25] was chosen to quantitatively assess the usability of the *Enable* app [26]. The MAUQ enables the usability assessment of mHealth apps from the user’s perspective. The MAUQ stand-alone version was formulated to evaluate 3 constructs of usability—ease of use, interface and satisfaction, and usefulness—as well as the overall usability score for the app through descriptive statistics. Each of the items of the MAUQ is rated on a Likert scale ranging from 1 (*strongly agree*) to 7 (*strongly disagree*), with the overall score ranging from 0 to 100. In addition, the questionnaires were complemented with a set of questions developed by the authors. Newly added questions concerned the use of other mHealth apps, smartphone ownership, sociodemographic information, and a free-text field to be able to describe the study sample more precisely. The target sample size was 100.

In addition to the questionnaire, open-ended, semistructured, and guide-based interviews with patients were conducted to explore their perspectives on the usability of the *Enable* app. The interviews were conducted by 2 female researchers (CA and LW) with a professional background in health services research and implementation science. Both researchers have profound experience with qualitative interviewing. The interview guide (Multimedia Appendix 1) was developed by a team of health services researchers (LW and JM) based on an extensive literature review and recommendations from the app developers. Afterward, the interview guide was pretested. This study is reported according to the COREQ (Consolidated Criteria for Reporting Qualitative Research) guidelines (Multimedia Appendix 2 [27]).

Furthermore, to objectively assess how patients interact with the app and identify potential usability issues, an eye-tracking study was conducted. The eye-tracking study was conducted by a usability expert (PM) and a team of health services researchers (CA and LW). A total of 5 tasks were formulated for the eye-tracking study (Multimedia Appendix 3): app log-in, filling in a questionnaire, searching and reading an article, and logging out from the app. To determine the comprehensibility of the tasks, the duration of the study, and the workings of the *Enable* app, 2 pilot tests were conducted. Following the pilot test outcome, the eye-tracking studies were carried out for 60 minutes with each participant, including the eye tracker setup and the retrospective interview.

The chosen mixed methods approach is designed to systematically collect, cross-validate, and analyze both qualitative data (derived from semistructured interviews and eye tracking) and quantitative data (obtained through the MAUQ). The inclusion of the eye-tracking method in the usability study enriches the capacity to integrate subjective and objective metrics. The qualitative aspect of the eye-tracking analysis enhances the understanding of the user’s app perception within the context of individual interactions and app usability. Simultaneously, semistructured interviews enable an assessment of the practicality of integrating the *Enable* app into daily routines. In contrast, the quantitative data derived from the questionnaire provide precise metrics related to usability measurements.

Hence, the mixed methods approach investigates the *why* and *how* aspects through qualitative inquiry, supplementing conventional quantitative and visual data analyses. The fusion of direct observations of user interactions with the app, poststudy retrospective interviews, semistructured interviews, and the usability questionnaire collectively supports the contextualization and comprehensive interpretation of the gathered data.

### Data Collection and Analysis

#### Quantitative Measures

The MAUQ and sociodemographic questionnaire were mailed twice to all patients after inclusion in the RCT. Data collection lasted from May 2021 to October 2022. Study data were collected and managed using REDCap (Research Electronic Data Capture; Vanderbilt University) tools [28] hosted at Heidelberg University Hospital. REDCap is a secure, web-based software platform designed to support data capture for research studies. After completion, all data were exported from REDCap to the R statistical software (version 4.0.4; R Foundation for Statistical Computing). All data were checked for completeness and analyzed by study team members. A descriptive analysis of the questionnaires was performed using R. Means and absolute and relative frequencies were calculated.

#### Qualitative Measures

Interviews were conducted after participants had used the app for 8 weeks. The interviews took place partly face-to-face at the clinic and by telephone in consideration of current guidelines for preventing infections with SARS-CoV-2 (ie, participants and researchers wore appropriate masks and distance was kept at all times). Nonparticipants were not present during the interviews. No relationship with participants was established before taking part in the study. No repeated interviews were conducted. No field notes were taken. All interviews were audiotaped, pseudonymized, and transcribed verbatim. Transcripts were not returned to participants for verification. Data were transcribed, managed, and analyzed using MAXQDA Standard 2020 (version 20.4.1; VERBI GmbH). After 16 interviews, data saturation was discussed among the researchers. As no new themes emerged in later interviews, the researchers agreed that data saturation had been reached and no additional interviews were necessary. After completion of data collection, thematic analysis of the data was conducted independently by 2 researchers (CA and LW) [29]. First, the researchers reviewed the transcripts independently and identified themes from the literature and the interview guide and inductively from the data. Second, discrepancies were discussed in iterative cycles until a consensus on themes and the final coding scheme was reached. All themes were organized into main themes and subthemes. Each theme was clearly defined by a quote from the interview transcripts (Multimedia Appendix 4). Quantitative and qualitative data were analyzed separately.

For the eye-tracking data collection process, an assigned room where the Tobii Pro Nano (Tobii AB) was installed at the hospital was used; the Tobii Pro Nano is an eye-tracking device specifically designed for small screens, including smartphones. This hardware features a sampling rate of 60 Hz, measures 17 × 1.8 × 1.3 cm, and includes a USB type-A connector. The Tobii Pro Nano was securely affixed to the mobile phone stand, and the *Enable* app was installed on a smartphone. To facilitate data capture, both the smartphone and the eye tracker were connected to a laptop running the Tobii Pro Lab software (version 1.194) via USB cables. For the purposes of this study, both an Android device (Samsung Galaxy 10, Android version 11) and an iOS device (iPhone 11, iOS version 14.6) were available to users. The choice of smartphone was contingent upon the user’s preferred operating system. The eye tracker recorded the participants’ interactions with the *Enable* app, such as task completion time, participants’ navigation, gaze plots, and heat maps [30-32]. A heat map was used when fixation duration data were collected [30,31], and a gaze plot was used when location of eye movement data were collected [33,34]. For this study, after the completion of tasks, the study moderators composed post hoc questions pertaining to the interactions, participants’ experiences, and usability issues observed during the procedure. The post hoc questions were discussed with the participants in a short debrief. The debriefing sessions were held to gather direct feedback from participants after interacting with the *Enable* app, allowing for a deeper understanding of the participants’ behavior and interaction with the app. Through these debriefing sessions, participants could provide context and commentary on their behavior and interaction [35]. Engaging users using post hoc questions, such as using images or live content from recorded sessions, allowed for a better understanding of the real-life context with minimal disruption as it facilitated the recall of situational information prompted by data, sound, or visual imagery.

The data analysis was based on the recordings of the study sessions concurrent with the eye movements of participants. The retrospective analysis involved transcribing participants’ feedback from the audio recordings obtained during the debriefing sessions. Data analysis also included the completion of predefined tasks by the participants, task completion time, and completion status of the tasks. The analysis focused on task performance analysis and the problem analysis of eye-tracking metrics and participants’ feedback.

### Ethical Considerations

The study was conducted in accordance with the Declaration of Helsinki and approved by the Ethics Committee of Heidelberg University Hospital (S-685/2020). All participants provided written informed consent for taking part, audio recording of the interviews, and video recordings during the eye-tracking procedures. Confidentiality and anonymity were ensured throughout the study. The data was protected against unauthorized access. No incentives or compensation was provided to participants for study participation.

## Results

### Overview

The MAUQ was sent to 165 patients recruited from the *ENABLE* RCT. The response rate was 63.6% (105/165) for the MAUQ at week 4 and 56.4% (93/165) for the MAUQ at week 20. A total of 105 questionnaires for the MAUQ at week 4 (including sociodemographic data) and 93 questionnaires for the MAUQ at week 20 were analyzed. In total, 16 patients were recruited for the interviews, and 10 were recruited for the eye-tracking procedure. The mean duration of the interviews was 25 (SD 7.34) minutes.

### Sociodemographic Characteristics

The sociodemographic data of the participants in the *ENABLE* usability study are shown in Table 1, and additional characteristics of the participants regarding smartphone and app use are shown in Table 2. The mean age of all participants (n=105) was 51.3 (SD 10.9) years.

**Table 1:** Sociodemographic characteristics of the participants.

Characteristic			Interview participants (n=16), n (%)		Eye-tracking study participants (n=10), n (%)		Questionnaire participants (n=105), n (%)
**Gender**							
	Woman	16 (100)		10 (100)		105 (100)	
**Age group (y)**							
	<30	2 (12.5)		2 (20)		2 (1.9)	
	30-40	2 (12.5)		1 (10)		16 (15.2)	
	41-50	6 (37.5)		3 (30)		32 (30.5)	
	51-60	4 (25)		2 (20)		33 (31.4)	
	61-70	1 (6.3)		0 (0)		16 (15.2)	
	71-80	1 (6.3)		0 (0)		6 (5.7)	
**Education**							
	Academic degree	9 (56.3)		6 (60)		37 (35.2)	
	High school education	0 (0)		0 (0)		13 (12.4)	
	Lower or intermediate secondary school	5 (31.3)		4 (40)		54 (51.4)	
	Prefer not to say	2 (12.5)		0 (0)		1 (1)	
**Employment**							
	Employed	11 (68.8)		9 (90)		66 (62.9)	
	Unemployed	0 (0)		0 (0)		17 (16.2)	
	Studying or vocational training	1 (6.3)		1 (10)		1 (1)	
	Retired	2 (12.5)		0 (0)		18 (17.1)	
	Prefer not to say	2 (12.5)		0 (0)		3 (2.9)	

**Table 2:** Additional participant characteristics on smartphone and app use.

Characteristic			Interview participants (n=16), n (%)		Eye-tracking study participants (n=10), n (%)		Questionnaire participants (n=105), n (%)
**Use of smartphone (y)**							
	≤10	9 (69.2)^a^		5 (62.5)^b^		56 (53.3)	
	>10	4 (30.8)^a^		3 (37.5)^b^		44 (41.9)	
	Prefer not to say	0 (0)		0 (0)		5 (4.8)	
**Use of other mHealth^c^ apps**							
	Yes	4 (28.6)^d^		5 (50)^e^		33 (31.4)	
	No	10 (71.4)^d^		5 (50)^e^		71 (67.6)	
	Prefer not to say	0 (0)		0 (0)		1 (1)	
**Frequency of app use**							
	Daily or several days a week	5 (45.5)^f^		3 (37.5)^b^		0 (0)	
	Once a week	5 (45.5)^f^		4 (50)^b^		0 (0)	
	Once a month or less	1 (9.1)^f^		1 (12.5)^b^		0 (0)	

^a^n=13.

^b^n=8.

^c^mHealth: mobile health.

^d^n=14.

^e^n=10.

^f^n=11.

### Quantitative Measures

The MAUQ [25] was used to collect quantitative data on the usability of the *Enable* app. The data were collected at weeks 4 and 20 starting from the baseline of the study. Quantitative data gathered from the MAUQ were analyzed using descriptive statistics. Only complete questionnaires for which the MAUQ score could be calculated were evaluated. Hence, 32.4% (34/105) of incomplete questionnaires collected at week 4 and 29% (27/93) of incomplete questionnaires collected at week 20 were excluded from the analysis. According to Zhou et al [25], the usability of an app is calculated based on the average of the responses to all statements. The higher the overall average, the higher the usability of the app. In this study, the overall usability scores for weeks 4 and 20 were 89.15 (SD 9.65) and 85.57 (SD 12.88), respectively. The mean for each of the subscales from week 4 to week 20 was also calculated and is presented in Table 3. The results show that the usefulness score declined over time from week 4 (80.89) to week 20 (77.33). In addition, the *interface and satisfaction* score also decreased but not as much as that of the *usefulness* subscale. The *ease of use* score, in contrast, remained constant at both weeks 4 and 20.

**Table 3:** Quantitative analysis of the mHealth App Usability Questionnaire and subscales.

Time point	Overall, mean (SD)	Ease of use, mean (SD)	Interface and satisfaction, mean (SD)	Usefulness, mean (SD)
Wk 4 (n=71)	89.15 (9.65)	92.41 (11)	91.6 (10.15)	80.89 (15.67)
Wk 20 (n=66)	85.57 (12.88)	92.27 (11.91)	88.23 (13.6)	77.33 (16.63)

### Qualitative Measures

#### Interviews

**Figure 2:**
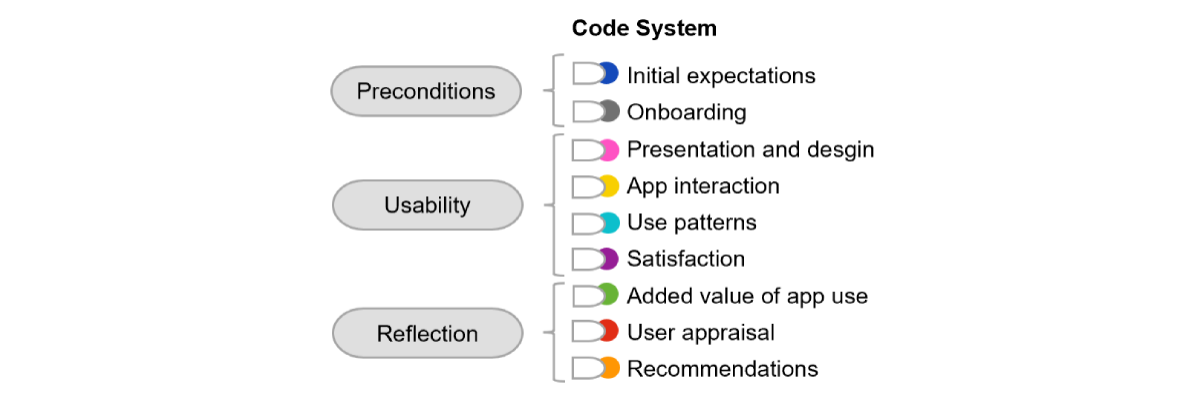
Overview of identified themes.

In total, 527 text passages were coded during the interviews. A total of 9 themes and 60 subthemes were identified, each of which could still be categorized under the superordinate themes of preconditions for app use, usability, and reflection. These themes are summarized in Figure 2.

#### Preconditions

##### Initial Expectations

As an opening question in the interview, patients were asked about their initial thoughts when they first heard about the *Enable* app. The most frequently mentioned expectations were related to the quality of information. Patients expected the information in the app to be updated regularly, understandable, and in line with the latest research. Another expectation was that the app would provide contemporary therapy support and be perceived as modern, including replacing printed brochures. Patients expected the app to provide guidance over the course of therapy, contact options, and easy access to relevant information. Approximately half of the participants had neutral expectations for the app:

Yes, I already thought that it [the app] would support me through everyday life and therapy, that I can also use it to organize myself a bit.

Interview 9; transcript position 2

##### Onboarding

The aspect of the onboarding process was not part of the interview guide. However, individual participants reported that they felt well supported by the study staff at the beginning of their app use. Even if they were initially overwhelmed by the app or experienced technical difficulties, participants expressed that they received the necessary support and were able to handle the app:

Oh dear, now I have to dig into yet another app. I don’t know if I can handle it. But the more I got a grip on it, the better it worked.

Interview 3; transcript position 2

#### Usability

##### Presentation and Design

Patients were asked to describe their impressions of specified design aspects. Overall, patients were content with the color scheme and perceived it as pleasant without being boring or flashy. For some patients, this cheerful esthetic contributed to a sense of joy when using the app and encouraged them to use it more often:

[The design is] very friendly. Very beautifully visualized. I always enjoy opening the app. It is also well designed, you always have the feeling that it is not draining in any way, it is more playful with all these images and visualizations. I find it very very clear.

Interview 11; transcript position 34

Regarding the layout of the written information, patients appreciated how the most relevant parts were highlighted through the positioning of boxes. The font size, design, and structuring of the information were seen as adequate. The selection of accompanying images was described as empathetic and not too explicit. The app included a personalized visual representation of the therapy progress. This display was also rated as clear and useful. Patients explained that the presence of this display motivated them:

I found this progress bar, which shows me how long I will be in therapy for, especially beautiful. It...motivated me, showing me that there is always a path forward and that the therapy will soon be over.

Interview 13; transcript position 43

##### App Interaction

Regarding usability, 4 important aspects emerged while interacting with the app. Neither the positioning nor the design of the app icons were perceived as entirely intuitive. However, patients grew acclimated to the icons, and thus, this did not further impede usability:

Yes, the icons that were down in this bar. In the beginning, I didn’t know the meaning of each icon. But when I took a closer look once, I knew it for the next time.

Interview 7; transcript position 39

Log-in and log-out procedures were described as easy and quick and did not pose any problems for the patients in this study. Most patients had no issues working with the app’s structure. They could easily navigate within the app and were able to find what they were looking for:

I found my way around the app really quickly. I haven’t tried all the features yet, I haven’t clicked on everything because I don’t need it all. But I have always been able to find the things that I wanted very quickly, and everything is right there when you click on it.

Interview 11; transcript position 44

Overall, patients liked using the app as it enabled them to access information *on the go*. Patients described that having their smartphones with them at all times allowed them to read information given the absence of other electronic devices such as laptops or tablets. However, a few patients mentioned the additional benefits of having a web-based version of the *Enable* app.

##### Use Patterns

This code encompasses descriptions of how and when patients used the app. Most patients experienced changes in the frequency of app use. In the beginning, they used the app often, and some patients used it multiple times per day:

In the beginning, shortly after my diagnosis, I had a lot of questions—for my physicians, how things work and so on. During this time, it (the app) really helped me a lot.

Interview 10; transcript position 24

Over time, use declined. This development was mostly due to lower demand for support and information as patients became used to therapy proceedings. Patients also used the app less as they felt that they had already read everything.

After this initial phase, patients reported using the app whenever they needed to look up appointments, had free time (eg, during waiting times before physician’s appointments), had or experienced new side effects from their treatment, were prescribed new medications, or were prompted by push notifications:

I always used it shortly before my [chemotherapy] appointments. Or when I had questions regarding diet and exercise. And sometimes there were questionnaires I had to fill in. And yes, as soon as the app said “there is news,” I opened it...And to look up times for my appointments.

Interview 10; transcript position 12

##### Satisfaction

Patients praised the general aspects of the app and liked the idea of having a digital tool accompanying them throughout their therapy; for example, the app provides a good overview of relevant topics, especially at the beginning of the disease. Except for 1 interviewee, all participants (15/16, 94%) would recommend the app to others:

...because it really provides a great overview...because so many aspects are addressed. Not only the type of therapy, but also just different things about cancer. Especially at the beginning these keywords—Yes, these terms in the boxes from tiredness to fatigue and polyneuropathy and different things.

Interview 7; transcript position 47

#### Reflection

##### Added Value of App Use

When asked about the concrete benefits of the app in everyday life, several aspects were mentioned. The most important aspect for the participants was the information on therapies and side effects, which was perceived as helpful, especially in the initial phase of therapy. The quality of the information was praised as the app’s information was considered understandable and its origin was considered reliable:

You feel informed, you feel—that gives you a form of security, because you say to yourself: Well, if I have the information from here [the app], then it was completely clear to me: I don’t have to look it up again. That’s true for me because these are reliable information providers who wrote this.

Interview 12; transcript position 81

The comprehensibility and language level were also perceived as adequate. Statements on the amount of information were heterogeneous according to individual information needs. However, the amount of information was predominantly perceived as sufficient in the context of the app. Furthermore, the appointment display, contact information, and progress bar were found to be helpful and clear. With regard to the contact information provided in the app, the fact that it was easy to find was rated positively.

Some patients reported that the questionnaires in the app gave them a positive feeling as they reflected on their condition and (in the intervention group) it was experienced positively that the questionnaires were read by the study staff and that staff could react proactively to them if necessary. Overall, patients perceived the app as a good therapy companion that guided and supported them through the various phases of the disease and therapies.

##### User Appraisal

Users’ opinions on the existing functions and features of the app were added to this category. Most patients complained about the appointment display as the date and time on the app did not always correspond to the actual clinic appointments (eg, in the case of last-minute postponements):

It’s a shame that the—I don’t know how the appointments displayed in the app, how often those are matched. I’ve had frequent differences there. Especially when appointments had to be postponed, the chronology was no longer correct for me.

Interview 9; transcript position 2

Regarding the quantity of information, some patients wished for more in-depth information or links to other information platforms. It was remarked that the amount of information available varied depending on the topic. Regarding the quality of the content, patients noted that the listed side effects or drugs were grouped differently. For instance, the patients were unable to locate paclitaxel as it belonged to the taxane drug class. In total, 12% (2/16) of the patients in particular perceived errors in spelling, punctuation, and grammar as distracting. The presentation of the contact information on the app was described as difficult to find, especially in emergencies. The additional pop-up notifications of the app updates were rated negatively as it was not apparent to the user what exactly was new in the app. Furthermore, respondents ascertained that the menu navigation was not intuitive enough and, therefore, needed to be improved.

##### Recommendations

Statements about features of the app that are not yet offered were classified as recommendations or wishes. Most wishes were mentioned in relation to the appointment display. Patients would like to have additional information about appointments, such as directions, a reminder function, the ability to export appointments from the app to private calendars (eg, Google or Outlook calendars), or the ability to make appointments directly from the app. The desire for self-administration (ie, areas such as appointments, questionnaires, or therapy progress that can be actively managed by the patient) was also frequently voiced. In addition, some patients wished to view the questionnaires that had already been completed to be able to monitor their condition over the course of therapy:

With the exception of filling in the questionnaires, you can’t work with the app yourself. Therefore, if you could manage things in the app by yourself, then of course I would think that would be great.

Interview 9; transcript position 2

Patients also wanted the content of the app to be updated, expanded, and adapted to new scientific findings. In this context, there was a desire for more explanatory videos to be included in the app. Patients also suggested that the app should offer more information about current and upcoming clinical trials for patients with breast cancer. To see what content in the app has already been read, patients suggested a read status, where content that has already been read is highlighted. Emergency contacts should also be highlighted in the app to make them easier to find, for example, by displaying them on the home page:

Especially the emergency numbers, I don’t know how to get something like that into the app, but that might be an idea, because I’ve been looking a lot for the right contact person. Maybe that would also be something that you could highlight a little bit or display as a button.

Interview 4; transcript position 32

To be able to find certain topics more quickly, the need for a search function was mentioned several times. Furthermore, to improve the readability of the content, patients would like to be able to adjust the font size. It was also suggested that the app could be used on other devices, such as tablets.

### Eye Tracking

#### Overview

**Figure 3:**
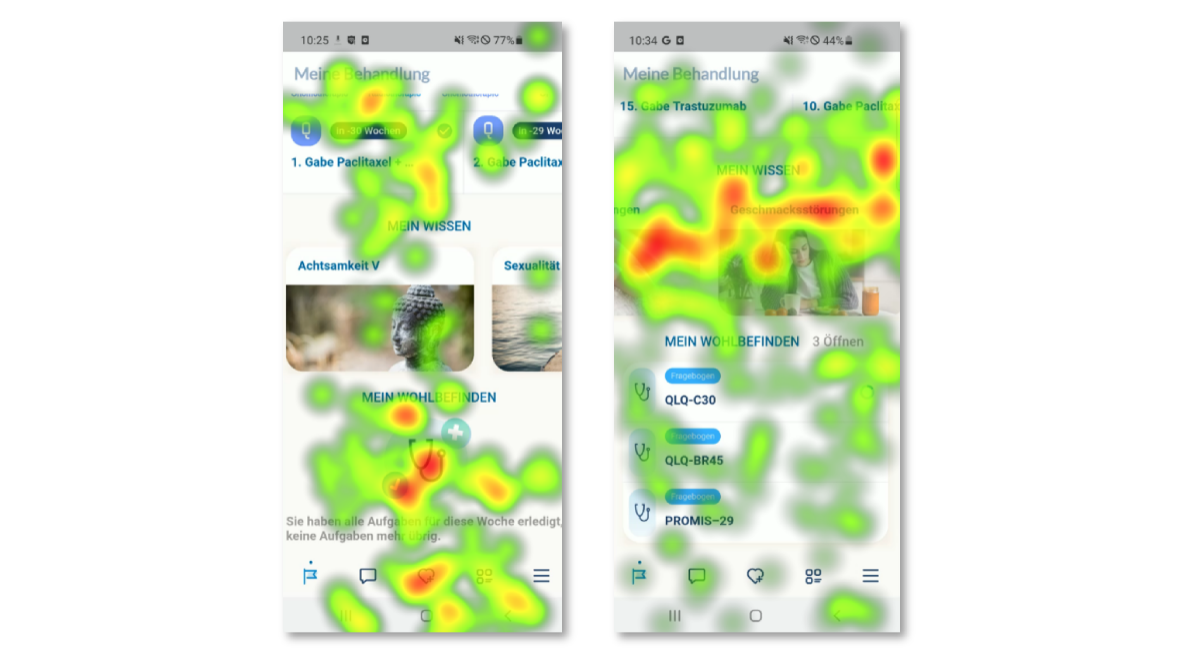
Heat maps from the eye-tracking analysis.

The analysis of the data collected from the eye-tracking recordings as well as the retrospective interviews showed that the participants found the app easy to use. We observed that most participants completed the given tasks, although the time taken to complete a few tasks proved to be challenging. On the observations and retrospective interviews during the eye-tracking study, we discovered 3 noticeable patterns related to the design and layout of the app, content and navigation through the app, and additional features the participants would like to have in the app. Figure 3 shows exemplary heat maps from the eye-tracking analysis. The data collected during the task performance, such as the task completion rate and task completion times, are provided in Multimedia Appendix 5.

#### Design and Layout of the App

Many of the participants had problems understanding and interpreting the icon at the bottom of the screen. The eye-tracking data showed fixations at the bottom of the screen while the patients clicked each of the icons displayed to view the content of the page. Patients expressed a preference for finding the most important information, such as appointment dates and the progress of a questionnaire, at the top of the screen. This finding indicates that patients expect important information to be located at the top of the app’s layout. Furthermore, the patients actively mentioned that the retrievability and visibility of the questionnaire were low. Although the questionnaires were available on the home screen of the app, patients believed that the questionnaires were available on the menu. In contrast, patients found the overall layout of the app to be acceptable.

#### Content and Navigation of the App

Regarding the content of the app, patients showed more interest in the titles of the articles (eg, topics such as symptoms or side effects) than in the images displayed. When asked during the retrospective interviews, patients mentioned that they did not pay attention to the images as they provided no information on what the article was about. Patients preferred to read the title of the article as it gave them information about its content, as shown by the red areas of the heat maps in Figure 3. Moreover, many participants explored the app to find the right information or icon to perform the tasks. However, this correlates with how frequently patients used the app. During the interviews, some patients said that they used the app frequently, for example, every day, to read articles on side effects or symptoms and fill out questionnaires regularly, whereas some patients used the app frequently at the start phase of the *ENABLE* RCT and later minimized the use of the app except to fill out questionnaires. The data showed that patients also had issues navigating through the app, especially related to the task of finding a specific article. Analysis of the recorded data of the participants’ navigation and gaze plots from the Tobii Eye Tracker showed that patients looked for a search function. Most patients clicked the menu icon; however, they did not proceed further to find the article nested under the *Symptoms* category on the menu. In addition, some patients searched for the article on the start page along with the other articles already displayed.

#### “Would Like to Have” (Wishes)

Participants identified a need for additional features in the *Enable* app as a consequence of the challenges they encountered during the eye-tracking study tasks. These suggested features were considered as *nice-to-have* options and were based on the specific problems faced by the participants during the study. The first was the availability of an option to mark an article as a favorite and be able to view the favorite article on the start page. Second, patients desired to have more articles or information about the symptoms and side effects of breast cancer and its treatments. Third, the icon currently representing contact information for health care providers (*My Care Team*) was misleading. Patients preferred to have another icon that indicates contact or communication as this would enable them to contact the study nurses more quickly. Finally, a search option was suggested by all participants.

## Discussion

### Principal Findings

The aim of this study was to investigate how patients with breast cancer rated their engagement with the *Enable* app, the user experience, and the benefits of using the app. In particular, the design, layout, navigation, content, and requests for new features were identified as important outcomes of interest for evaluating the app and further improving it to meet user needs. The interviews provided valuable suggestions for optimizing the app and the implementation process. The design and color scheme were rated very positively overall. In terms of use patterns, it was noticeable that the frequency of app use decreased over the therapy period.

Patients found the app easy to navigate. However, there was some criticism that the menu icons were not intuitive enough, especially at the onset of use. Perceived benefits were discussed extensively in the interviews. Patients found the information on therapies and side effects very useful. The appointment display and progress bar were also found to be helpful and motivating. At the same time, the appointment display was most often criticized, and it was the feature for which there were the most recommendations for change (eg, to be able to manage appointments autonomously in the app or set reminders). In terms of content, it was mentioned that there was a lot of information on some topics and not enough on others. Patients also wanted more content updates within the app (eg, on current topics such as the COVID-19 pandemic) and a search function to access specific content.

A study by Ansaar et al [36] showed that nearly 78% of all usability evaluation studies in their systematic review used a questionnaire-based method. However, using mixed methods approaches in usability evaluation studies provides benefits such as the possibility to balance the advantages and disadvantages of the different methods. Moreover, by applying the mixed methods approach, both subjective and objective aspects can be combined to assess usability [36]. In many aspects, such as the *navigation*, *recommendations*, and *perceived benefits* codes, the results of the different survey methods support each other. However, the interviews and eye-tracking study sometimes provided different findings. For example, the importance of images within the app was positively highlighted in the interviews. In contrast, the eye-tracking study and retrospective interviews revealed that images played a subordinate role for patients, with titles being more important for finding relevant content in the app. Although participants reported in the interviews that they were able to navigate easily within the app and find the content they were looking for, we observed in the eye-tracking study that there were difficulties with finding specific content. Furthermore, the interview inquiries primarily centered on the practicality of incorporating the *Enable* app as a follow-up intervention in daily life. Meanwhile, the use of eye-tracking technology allowed for direct, real-time observation of user behavior while engaging with the app through task performance. Despite patients reporting the ability to regularly use the app without difficulty, the eye-tracking study’s direct observation unveiled valuable insights into their actual use patterns within their everyday routines. In this context, disparities between the results obtained from the 2 methods emerged, possibly stemming from users’ lack of awareness regarding any issues until they were prompted with specific inquiries.

### Comparison With Prior Work

Our results on the MAUQ indicate good usability. The results for the total scale showed that usability decreased from weeks 4 to 20. A decrease in usability over time has also been observed in previous studies [37-39]. Possible explanations for this decline in our study can be found in the interviews, indicating that the extent of app use also decreased over the course of therapy. Patients found the app to be particularly advantageous at the start of their therapy because of their great need for information. However, as they gained more knowledge about the disease and its treatment, their demand for information decreased. In addition, patients reported that the app lost its appeal once all the available articles had been read, often leading to a desire for new content to be added. Patients also expressed a need for additional features or improvements as they continued to use the app. As a result, the decrease in the app’s usability score could be attributed to patients perceiving it to be less useful after an extended period of use owing to the lack of content updates and unmet desires.

Looking more closely at the subscales of the MAUQ, *usefulness* had the lowest score compared with *ease of use* and *user interface and satisfaction*. These items assess whether the app is helpful and useful for patients’ health and well-being. This relationship is also apparent when looking at the *usage patterns* category from the interview analysis. It appears that patients are less likely to use the app because of the lack of new content. This is consistent with the findings of other studies on mHealth apps for patients with breast cancer [16,40,41]. As an implication for similar apps for other chronic conditions, it seems important to update the app content on a regular basis to provide patients with an incentive to continue using the app as well as strengthening patients’ satisfaction and information needs. Consistent with the findings from the interviews and eye-tracking study, only the *ease of use* subscale remained almost stable over the duration of app use.

In the context of other usability studies on mHealth apps, the importance of paying more attention to the user group of older adults is emphasized. The different age ranges of patients and the different levels of technical affinity for older patients are mentioned as possible factors causing usability problems. Some studies emphasize that these factors are often overlooked and need to be considered when developing mHealth apps [42,43]. In our study, these aspects were less evident. With an average age of 51 years, our study participants do not represent a predominantly older population but are close to the German population average for women, which is 46 years [44]. In contrast, the study participants were also far below the average age of 64 years for patients with breast cancer. Therefore, further research on app development and usability with a focus on older participants should be conducted to more adequately represent the typical population of patients with breast cancer.

Considering the preferred device for using the *Enable* app, most participants were content with using the app on their smartphones. However, there were isolated requests to be able to increase the font size of the content and use the app on a larger-scale device, such as a tablet or PC. This issue was also mentioned by participants in a usability study by Jessen et al [45], in which an mHealth app for self-management of chronic diseases was evaluated.

Although the onboarding process was not part of the interview guide, some patients actively recalled how they were introduced to the app as well as how they perceived the technical onboarding process. The patients did not experience issues with these steps and reported being content with the process, mostly because of the strong support of the study team. Previous research has pointed out that complex registration and log-in procedures can be perceived as especially cumbersome by patients and can lead to stopping app use [46-48]. Our study identified the strong interpersonal connection with and continued support from the study team as a positive influence on the perceived ease of onboarding. This support took place in the context of a research study and is not viable in a real-world implementation. However, the issue of technical support arose exclusively during the qualitative interviews. We did not collect any quantitative data on this topic. Thus, further streamlining of the onboarding process while being mindful of health care workers’ limited time resources should be an area for future research.

### Strengths and Limitations

The chosen mixed methods approach can positively support the further development of the app. The expansion of the classic social science method spectrum to include technical methods such as eye tracking made it possible to combine the subjective patient perceptions reported in interviews and questionnaires during everyday use with objective measurements under laboratory conditions.

However, the integration of qualitative results and the objective measurement from the eye-tracking procedure introduced discrepancies. As noted previously, interviewees appreciated the use of images in the app, whereas eye-tracking results showed that more time was spent on the article titles than on the images. Another example is that the interviews and the questionnaire produced good ratings of usability, but the eye-tracking study showed that patients found it difficult to find defined content. Although difficult to analyze, these discrepancies are common in mixed methods studies [19]. In our study, these discrepancies could be explained by methodological differences. For example, reading a title naturally takes longer than glancing at an image, leading to a long fixation time. Therefore, this result does not allow for the conclusion that titles are more important than images. Here, the qualitative interviews were helpful in interpreting this finding. Regarding the second example—overall good usability scores in comparison with eye-tracking times—several interpretations appear plausible. First, it is possible that social desirability led patients to rate the usability more favorably in both the interviews and the questionnaire. Consequently, the objective measure via eye tracking revealed that usability was worse than in subjective measures. Second, the setting of the eye-tracking procedure (eg, unusual or uncomfortable sitting position, being observed by ≥2 researchers, or using a different device) could have led to changed patterns in (app use) behavior. Although we acknowledge these discrepancies, we conclude that the mixed methods approach and its results deepened the understanding of the studied topic and produced valuable insights, with discrepancies leading to vigorous and fruitful discussions among the researchers.

However, the generalizability of the study results is limited by several factors. To ensure that patients with lower digital health literacy could participate in the quantitative data collection without constraints, we decided to use printed surveys sent by mail. Patients returned them at their discretion. Hence, it cannot be verified whether the surveys were filled out at the correct time. In addition, some values were missing from the returned surveys, and manual data entry could have led to documentation errors. Incomplete or inconclusive questionnaires had to be completely excluded from the analysis as it was not possible to calculate the score. Although all necessary steps were taken to ensure high-quality and reliable data (eg, data entry was always checked by another researcher), using a web-based survey instead of a printed survey could have made data collection easier, faster, and more reliable. These trade-offs have to be balanced in future research projects.

This study population contained an above-average proportion of academics, especially among the subgroups of interviewees and eye-tracking study participants. This should be taken into account when interpreting these results. A systematic review by Niazkhani et al [49] showed that patients with lower educational attainment and limited health literacy were less likely to intend to use an electronic patient health record and were more likely to use it ineffectively. Moreover, previous experience with computers or health technology has been associated with increased acceptance, and acceptance increases with higher education [7]. Although these results refer to electronic health records, they indicate that this aspect should be further investigated in future studies. Given the median age at breast cancer diagnosis of 64 years and the relatively younger median age of this study cohort, conclusions from this study must be interpreted with caution as they may not represent the views and digital literacy of older women with breast cancer [50].

The *Enable* app was developed specifically for patients with breast cancer. Consequently, our study sample included only female patients with breast cancer. Some of our results and recommendations may have limited generalizability to other patient populations. Nevertheless, we think that aspects such as the relevance of content updates, the accuracy of displayed appointments, or the intuitiveness of the app navigation might also be relevant beyond the target group. This should be verified in further research.

As part of the *ENABLE* RCT, reasons for dropping out were documented where available. These reasons were examined to see whether there were any indications of usability problems. A small proportion of the included study participants in the RCT dropped out because of physical exertion or feelings of being overwhelmed by the app. In this respect, further research is needed to understand how patients in later stages of the disease or with greater disease burden perceive the usability and benefits of the intervention. Furthermore, mHealth apps should be designed to be usable and helpful for these patient groups as well, especially in the context of patients living with cancer. As the mean age of participants in this study was relatively low, it can be assumed that there is a risk of selection bias. It is possible that younger patients decided to participate in the study and use the app because of a higher affinity for smartphones [11].

In addition, using the eye-tracking device led to further limitations. Potential participants in the eye-tracking study had to undergo an additional screening process to exclude patients wearing bifocal glasses. Although patients were recruited for the study, this criterion did not allow us to cast a wider net for the participant recruitment process. Furthermore, we also had the challenge of asking patients to sit still so that the eye-tracking data could be captured without breaks. However, this request is generally against the natural way in which users sit and interact with mobile devices. Another point to note is that the execution of the tasks on the app by the patients was deviated as the tasks were presented on paper and this retracted some of the gaze points of the patients. This is, in general, a common problem when tasks are not integrated into mobile apps during development for testing purposes.

### Conclusions

The results of this usability study demonstrate good usability of the studied app and potential for purposeful development. The design and color scheme were rated very positively overall. However, there was some criticism that the menu icons were not intuitive enough, especially at the onset of use. Noticeably, the frequency of app use decreased over the therapy period. Perceived benefits of the app were information on therapies and side effects. The appointment display and progress bar were also found to be helpful and motivating. Still, participants offered recommendations for changing the appointment display (eg, to be able to manage appointments autonomously in the app or set reminders). In terms of content, it was mentioned that there was a lot of information on some topics and not enough on others. Patients also wanted more content updates within the app (eg, on current topics such as the COVID-19 pandemic) and a search function to access specific content. The interviews and eye-tracking study revealed valuable suggestions for improvement as well as requests for additional app features. An important point is that the app currently provides information to the patient mainly passively. The patients’ wishes indicate that the app needs to be further developed so that they can actively enter information into the app and work with it. The overlap between decreasing usability and decreasing usefulness also suggests that the app needs to be regularly updated with new content to maintain its usefulness over time. These findings will be incorporated into the further development of the *Enable* app. We concluded from patients’ feedback and requests that similar mHealth apps could benefit from giving patients a more active role (eg, being able to actively document side effects as they show up instead of being prompted to do so). In addition, regular updates to app content (eg, adding new informational pieces) could further contribute to and, thus, encourage the continued use of mHealth apps.

## Appendix

Multimedia Appendix 1 Interview guide.

Multimedia Appendix 2 COREQ (Consolidated Criteria for Reporting Qualitative Research) checklist.

Multimedia Appendix 3 Eye-tracking tasks.

Multimedia Appendix 4 Definition of themes.

Multimedia Appendix 5 Task performance data.

## Abbreviations

COREQ: Consolidated Criteria for Reporting Qualitative Research
ISO: International Organization for Standardization
MAUQ: mHealth App Usability Questionnaire
mHealth: mobile health
PRO: patient-reported outcome
QoL: quality of life
RCT: randomized controlled trial
REDCap: Research Electronic Data Capture

## Data Availability

The data sets used and analyzed during this study are available from the corresponding author upon reasonable request.
