# Supplementary material for: Usability and User Experience of an mHealth App for Therapy Support of Patients With Breast Cancer: Mixed Methods Study Using Eye Tracking
Source: JMIR Hum Factors. 2024 Mar 5;11:e50926. doi: 10.2196/50926 (PMC10951836; doi:10.2196/50926)
Supplement: Multimedia Appendix 1 [file humanfactors_v11i1e50926_app1.docx]

**Appendix 1: Translated interview guide**

**Introduction:**

The “ENABLE” app was developed to support breast cancer therapy. What were your initial thoughts on this idea?

What do you think about this concept now, in general, after having used the app?

**Main interview questions:**

Please think back to the time when you started using the app.

- What did you expect from the app?
- To what extent were these expectations met?

How often do you use the ENABLE app?

- (follow up question if used seldomly): What should be changed in the app so that you would use it more often?

To what extent does the app support you throughout the therapy? (e.g., overview of appointments, mobile lexicon, information)

Which functionalities do you find most helpful?

Which functionalities are missing in the app?

How did the information in the app help you?

The hospital you are being treated at also provides other kinds of support. To what extent did the app encourage to take advantage of these?

Thinking about the app’s design: how do you perceive the design?

- (possible items for follow up questions): e.g., positioning of images and text elements, perception of the colors, font…
- (follow up question if design was perceived negatively): what would you like to change regarding the design?

Thinking about navigating the app: how intuitive was using the app for you?

To what extent were you able to navigate within in the app?

- (follow up question): Which aspects were especially easy? Which aspects were especially difficult?

How comfortable was reading app content on a smartphone display for you?

- (follow up question): How do you perceive the font size and the amount of text elements?

To what extent would you recommend the app to other patientS?

- (follow up question): Why? Why not?
- How would you rate the app on a scale from 1 (lowest score) to 5 (highest score)?

I am done with my questions regarding the ENABLE app. Do you have any further recommendations or ideas regarding the ENABLE app?

Thank you so much for the conversation and your interesting opinions!
