# Supplementary material for: Usability and User Experience of an mHealth App for Therapy Support of Patients With Breast Cancer: Mixed Methods Study Using Eye Tracking
Source: JMIR Hum Factors. 2024 Mar 5;11:e50926. doi: 10.2196/50926 (PMC10951836; doi:10.2196/50926)
Supplement: Multimedia Appendix 3 [file humanfactors_v11i1e50926_app3.docx]

**Appendix 3: Eye-Tracking Tasks**

**1. Log In**

Please open the Enable app and log in with the following login data::

Username EN90732

Password Eyetrack

PIN ****

- *The user can open the ENABLE app and login on his/her own.*

**2. Start page**

Please browse through all the articles currently highlighted on the start page and open one of the articles displayed.

- *The user can navigate to the start page and operate the list of currently highlighted items.*

Please go back to open and fill in the questionnaire QLQ-30.

- *The user can exit an article that has been opened.*

*The user can open and fill in the desired questionnaire.*

**3. My care team**

Find the contact details of your study nurses.

- The user is familiar with the "My Careteam" category in the menu and will find the contact details there.

**4. My content**

Please find the article "Heartburn" among all the contents of the app.

- The user is familiar with the content overview and can navigate through the higher-level categories.

The user finds the article "Heartburn" in the superordinate categories.

**5. Logout**

Please log out of the app now.

- The user is able to log out from the app.
