# Supplementary material for: Usability and User Experience of an mHealth App for Therapy Support of Patients With Breast Cancer: Mixed Methods Study Using Eye Tracking
Source: JMIR Hum Factors. 2024 Mar 5;11:e50926. doi: 10.2196/50926 (PMC10951836; doi:10.2196/50926)
Supplement: Multimedia Appendix 4 [file humanfactors_v11i1e50926_app4.docx]

**Appendix 4: Definition of themes**

| **Subthemes** | **Definition** | **Text Samples from transcripts** |
| --- | --- | --- |
| **Initial expectations** | | |
| Contemporary therapy support | Expectation that the app will be a modern therapy support tool in terms of both technology and sustainability. | *So I thought it was a great idea and absolutely modern and actually I have to say it was surprising that it hadn't got that far yet, but it was just the thought: Yes, it fits the times, it also fits me as a young patient, of course, and the fact that many more people are using more and more apps and devices like that and yes, I thought it was a good thing, that's why I agreed to it.* (Interview 6, TP 2) |
| Therapy guidance | Expectations regarding the app as an accompanying tool throughout the course of therapy. | *Yes, I already thought that this would support me through everyday life and therapy. That I can also use it to organize myself a bit.* (Interview 9, TP 2) |
| User-friendliness | Expectation that the app will be easy to use. | *Of course also about the - how easy that is to use in terms of user-friendliness, though - those are exactly the two things I expected to get.* (Interview 6, TP 8) |
| Contact options | Expectation that the app can be used to provide contact information and to establish closer contact with the treatment team. | *I was told in particular that I can find questionnaires on the app in the meantime to find out how I'm doing, and if I'm not feeling that good, that I'll also have a contact person there, or that I'll be contacted occasionally. That you are a bit more connected. And that was a little bit what I expected*. (Interview 11, TP 10) |
| Access to information | Expectation to receive therapy-relevant information through the app. | *I just thought that you could practically look up things you don't know or you can type in keywords or maybe you get information that you don't have in your mind*. (Interview 16, TP 10) |
| Neutral expectations | No specific expectations regarding the app. | *No, quite honestly, I joined with low expectations. I thought, let's see what it is, maybe it makes sense to participate. And it's not that much work. And let's see if it brings me an add-on or something. I didn't have a very clear idea of what it was supposed to deliver for me.* (Interview 8, TP 12) |
| Quality of information | Expectations regarding the linguistic preparation, topicality, and reliability of the information. | *Exactly, so yes, if you think about the basic idea of such an app, then I already expected that a few side effects and information for the individual medications are included. Of course, you don't know beforehand how it's going to be prepared, that's a very important point, how it's going to be prepared individually and in terms of language and scope* (Interview 6, TP 8) |
| ***Onboarding*** | | |
| Contact with study staff | Descriptions of how the study staff were involved in the onboarding process. | *It was explained to me how it works, by the doctor at the initial consultation, how it works and everything. And then I also received forms from a lady with a password.* (Interview 10, TP 6) |
| Overload with information | Descriptions of high initial demand to get to know the app | *First I thought “Oh dear, now I have to dig into yet another app. I don't know if I can handle it”. But the more I got a grip on it, the better it worked*. (Interview 3, TP 2) |
| Positive experiences | Positive experiences in relation to the on-boarding process. | *The doctor who informed me at the time gave me a short briefing about the procedures for my diagnosis and the therapy and I liked that and I accepted it. And then I waited to see how it would develop and then I was also informed accordingly and then that was set up and then I got the app and yeah, then it was pretty good.* (Interview 1, TP 6) |
| Difficulties | Difficulties experienced in relation to the on-boarding process. | *At first I didn't know that I would get an additional password. I tried and tried, but somehow it didn't work. And then I was given the additional password and it was explained to me how it works. And then I finally got it right.* (Interview 10, TP 6) |
| ***Presentation and Design*** | | |
| Color scheme | Statements on color selection and effect on app use. | *Great. It appealed to me right away. I am not quite a fan of pinks or roses, but I found it pleasant, just cheerful, that it is a cheerful color. It is also not pink in the sense, but it is yes salmon colors, so that works. That's still... So I found it aesthetically good and I also found it restrained in terms of style, not so flashy, but not boring gray either.* (Interview 12, TP 45) |
| Graphic design | Statements on text formatting, font size, or arrangement of content. | *[…] but otherwise - I find the ratio of image and text perfectly fine. Also certain things are then again emphasized by such a box, which are even more important*. (Interview 7, TP 33) |
| Display of the progress bar | Statements regarding the perception of the design of the progress bar. | *So, positive things I have to mention again, because I found it really great - these icons at the top, where you can see the progress of the therapy [...] That was really awesome - so that it almost has kind of a psychological effect.* (Interview 6, TP 20) |
| Pictures | Statements on the selection of pictures and their effect on app use. | *The selected pictures were also quite good - I don't want to say they were empathetic, but they were chosen in a way so I felt comfortable with the choice of colors, with the choice of pictures.* (Interview 6, TP 50) |
| General presentation | Unspecific statements on various design aspects of the app. | *Quite friendly. And very nicely visualized. So I always enjoy going through it. It's also well done, so you don't get the feeling that it's somehow exhausting, but instead a bit playful, due to the images and the visualizations. I find it very very clear.* (Interview 11, TP 34) |
| ***App interaction*** | | |
| App icons | Statements relating to the comprehensibility of the icons or symbols in the app. | *Yes the icons that are given are also down in the menu bar. So at the beginning you don't know what's behind the symbol, but when I look at it once, then I know the next time.* (Interview 7, TP 39) |
| Login and logout procedure | Experiences with the login or logout procedure of the app. | *Login also worked really well, it was also easy*.  (Interview 11, TP 44) |
| Advantages of smartphone use | Experienced benefits of app use on the smartphone in terms of being able to access information flexibly and quickly. | *Yes, because I always carry it with me, I'd say, I can just look it up quickly if I want to, and yes, well, if it's too small, I can also zoom in, so I think it's ideal that way - because it's available any time. You always have your phone with you.* (Interview 7, TP 41) |
| Navigation | Statements that the navigation and structure within the app was experienced as clear and intuitive. | *I found my way around the app really quickly. I haven't tried all the features yet, I haven't clicked on everything yet because I don't need it all. But I have always been able to find the things that I wanted very quickly, and everything is right there when you click on it.* (Interview 11, TP 44) |
| ***Usage patterns*** | | |
| Decreasing use over time | Description that the app usage decreases throughout the course of therapy. | *Of course you use it more in the beginning, I'd say. And once you've explored what's most interesting to you, you don't use it as much.* (Interview 7, TP 3) |
| Situation-related use | Descriptions of different situations in which the app is used. | *Always before my appointment. Almost every week, actually. Or when I have questions about nutrition or exercise, for example. Sometimes there are questionnaires that you have to fill out. And yes. As soon as it says "there is something new", I open it (the app). And otherwise every week when I have appointments, to see if I had the exact time in mind*. (Interview 10, TP 12) |
| Frequent or regular use | Descriptions of regular or frequent use of the app. | *I used it (the app) at the beginning - much more frequently, partly - yes five to ten times a* day (Interview 6, TP 18) |
| Redundancy or irrelevance of content | Information was considered irrelevant by users because they had already received this information via brochures, for example, or because the offered courses did not take place due to Covid-19. | *Well, in my case, I had already received all the brochures in advance, which I had read myself before the study started. And therefore, of course, there was a lot of information in there that I had already read in the brochures. - So there it doubled. You need to see how you can avoid duplicating the information. Because if you already know everything from paper formats, then the need to read it digitally is obviously a bit reduced.* (Interview 9, TP 2) |
| ***Satisfaction*** | | |
| Positive statements | Overall positive statements about the app. | *[...] because it really gives a great overview and if you just..., because many aspects are addressed. So not only the type of therapy, but also so many different things around the topic of cancer. - So just at the beginning these keywords - Yes, in the boxes these terms that you then just yes from fatigue to so fatigue and polyneuropathy and different things....* (Interview 7, TP 47) |
| Negative statements | Overall negative statements about the app. | *Well, one thing was - I simply had an individualized treatment path and the app was too general for me. So three quarters of the information was more of an overview of "What is the topic of cancer today", that's how I would summarize it, that's not supposed to sound so simple, but for the start it's important to deal with it, but at some point it has to be more individualized for me.* (Interview 12, TP 6) |
| Further recommendations | Statements about the extent to which participants would recommend the app to other patients. | *Yes, I would recommend it. Simply because - you get the information you're looking for, the information you need. Targeted.* (Interview 14, TP 50) |
| ***Added value of app use*** | | |
| Display of the progress bar | Statements on the experienced added value and impact of the display of the progress bar. | *Yes, first of all you get a little overview of where you are in the therapy. What's next, how far you have to go, I would say.* (Interview 7, TP 15) |
| Therapy support | Descriptions of how the app usage supported the participants over the course of therapy. | *And that is I think, in the situation in which you are, that is just an exceptional situation - it is - really a very very good companion and very detailed and very understandable too.* (Interview 1, TP 12) |
| Perception of the offers | Statements about the perception and the perceived added value of the additional support offered in the app. | *Exactly! Alternative medicine or what you can do or take in addition to the chemotherapy. So I thought that was not that bad. Just like nutritional counseling or something, so you have all kinds of phone numbers, so I think that's nice. That's helpful in any case.* (Interview 2, TP 44);    *Yes, I have taken advantage of the nutritional counseling. Yes, but as I said, also in advance with the information from the brochures. I had already initiated everything at the very beginning of the therapy.* (Interview 9, TP 2) |
| Questionnaires in the app | Statements on the perceived added value and impact of the questionnaires in the app | *And I also thought it was nice to be asked every week, "How are you today? Just that someone asked. But also to ask myself: How am I feeling right now?* (Interview 3, TP 2) |
| Contact information | Statements about how the app provides access to contact persons and what added value this has for the patients. | *Of course I also got calling cards and everything beforehand and information, but somehow with the app you just have it all together and if you are looking for something, I never went to the folder, but instead just looked it up in the app and made a call*.  (Interview 2, TP 46) |
| Information on therapies and side effects | Utilization and perceived added value of the information provided on therapies and side effects in the app. | *It was very helpful to me at the beginning, because when I had a concern, I actually looked up what is advised, especially with the side effects, because I thought: Okay, maybe there are a few tips and tricks - that might be chemo-specific, that I don't know at all. That was very helpful.* (Interview 4, TP 20) |
| Appointment display | Positive experience with the appointment function. | *What's really cool, or what I like very much, is that my appointments, which I have, are also displayed with the exact times. And if something changes, that's also entered. And that I also have an overview.* (Interview 11, TP 12) |
| Amount of information | Perceived added value regarding the scope or amount of information. | *Yes - yes I think you simply need the scope of the text, because otherwise you just have to inform yourself properly or to get the right information, so I find it well-balanced or not too much.* (Interview 7, TP 31) |
| Quality of the information | Statements on how the information was considered understandable and reliable regarding its origin. | *You feel informed, you feel - it also gives you a kind of security, because you say to yourself, well, now that I have the information, for example, if I have researched and found information or issues in Enable, then it was so clear to me that I don't need to look it up again. That's right for me, because these are reliable information providers who wrote this.* (Interview 12, TP 81)    *I also liked the language, so it wasn't a very simple language, where you feel like you're being made fun of as a layman, a medical layman, but at the same time it wasn't the kind of language where I need a dictionary. It was appropriate.* (Interview 12, TP 29) |

| ***User appraisal*** | | |
| --- | --- | --- |
| Navigation | Statements about experienced difficulties with navigating within the app. | *Clearer, so I said this swiping from right to left, that - is for me, well, that on the smartphone is unusual for me that I have to swipe for so long to get further in the flow and also the menu selection is in the lower is the unclear that - there.... So I didn't expect it like that [laughs].* (Interview13, TP 60) |
| Appointment display | Criticism on the presentation and correctness of the appointment display in the app. | *However, it's just a shame that the - I don't know how the appointments are managed in the app, how often they're matched, because I often had discrepancies there. Especially when appointments had to be postponed. - Then the chronology was no longer right for me and then I received reminders that something was scheduled even when nothing was scheduled at all.* (Interview 9, TP 2) |
| Quality of information | Criticism on the quality of the content regarding the sufficiency and comprehensibility of the available information (concerning medications, side effects, therapies). | *By the way, on the topic of Port catheter, there is not enough content or no content at all, I don't know. I think too little and there are two or three things that are just so banal. [...] So for me the topic of port catheter was important.* (Interview 12, TP 27) |
| Amount of information | Criticism on the depth or scope of information of the available content. | *Sometimes it was - a bit strange at the beginning, when you retrieved something, that for some things there was only very little information, i.e. very short information text, and for others quite a lot [...]* (Interview 6, TP 52) |
| Correct spelling and formatting | Criticism regarding spelling, punctuation, and inconsistent formatting of content. | *There are, well, there are -- yes, with information or with texts, that is already noticeable or punctuation, where you then have to read the sentence twice again and then say: Ah well, now I get it.  (Interview 7,* TP *33).*    *Partially there were still formatting errors in there.* (Interview 6, TP 52) |
| Pop-ups and news in the app | Criticism on presentation or visibility of news in the app. | *The only thing that irritated me sometimes was that the -- Enable app sent a notification to the smartphone that there was something new and it was - not a questionnaire, but I had to look first, where is something new now and (incomprehensible), then I was just a bit irritated.* (Interview 6, TP 22) |
| Contact information | Criticism on the presentation and sufficiency of contact information in the app | *What comes to mind spontaneously, I don't think I've seen it yet, maybe I just didn't notice it, maybe you can find other contact persons if you need them. So that you may have that directly in the app, [...] If you still have questions - that you have to call there.* (Interview 11, TP 40) |
| ***Recommendations*** | | |
| Appointments | Desire for additional features to the existing appointment function |  |
| Export appointment informations |  | *I just thought about the calendar feature, that you could connect the appointments with the calendar feature of Google or any calendar, that you could somehow create a connection.* (Interview 13, TP 18) |
| Appointment reminder |  | *Yes, yes. I mean, if you would link it with the Google calendar or whatever you use, then you have this reminder function - that would include that, but yes, if not, you could just expand the other - or with the appointments: Tomorrow is your next appointment or so. I don't know, such an announcement --* (Interview 6, TP 42) |
| Additional information on appointments |  | *Maybe detailed directions? So for example, if you click on the appointment, if you have an appointment, for example, clinic, control center 2 or something, and then you could still click on it and you would see everything a bit more in detail.* (Interview 11, TP 22) |
| Adjustment of the display | Desire for an option to customize the size of the app's content. | *But yes, I'm getting older too, my eyes aren't getting any better. ( incomprehensible ) And the font is a bit small. So if it could be larger, (that would be good).* (Interview 14, TP 84) |
| App use on other devices | Desire to use the app on other portable devices such as a tablet. | *I do not know whether you could also download the app on an iPad or something? Well, maybe that would also be a possibility, then you could read it in a larger format.* (Interview 13, TP 50) |
| Videos | Suggestion to integrate more explanatory videos into the app. | *Exactly. Maybe how chemotherapy works, in a 2 or 3 minute video. I could well imagine that.* (Interview 11, TP 56) |
| Read status | Desire to highlight content that has already been read. | *As I said previously, you can probably make a note somewhere that you have already read certain knowledge articles. That you see that, that you have already read that*. (Interview 9, TP 2) |
| View completed questionnaires | Desire to view questionnaires that have already been completed. | *I think that would be quite good. So that I can also see for myself how I was doing then and how I feel today. Roughly speaking. I don't know - there are always several sides. I don't know, maybe you would have a little better control over your state of mind for yourself.* (Interview 9, TP 2) |
| Self-administration | Desire that fields like appointments, questionnaires or therapy history can be actively managed by the app user. | *Except for filling out the questionnaires, you can't work with the app yourself yet. Therefore, if you could manage things yourself a little bit in the app, then of course I would think that would be great.* (Interview 9, TP 2) |
| Search function | Desire for a search mask to be able to find specific content in the app. | *I'm not even sure if there is a search function at the top of the medicines. If there isn’t, I'm sure I'd like to have it. Of course, so that I can go in with a few - yes, that I don't have to use Google now, but that I can type in a little shortcut and it gives me suggestions. Yes, that's what I'm looking for. And so on. But I would specifically like to search for drugs. Or information on side effects. Yes.* (Interview 8, TP 38) |
| Emergency contacts | Desire to highlight contact information and telephone numbers for emergencies. | *And what I also think, especially with emergency numbers, I don't know how you can get something like that in there, maybe that would be another idea, because I've been looking for the right contact person, maybe that would also be something that you could somehow highlight a little bit or create a button.* (Interview 4, TP 32) |
| Offers for participating in other studies | Suggestion that further opportunities for study participation could be referenced in the app | *and also with studies, as I said, I would also like that, because I searched a lot at the beginning to find out what was being offered, and it's not that easy*. (Interview 4, TP 26) |
| Regular updates/ News Feed | Desire that content of the app to be updated and enriched as well as adapted to new scientific findings. | *Maybe add more current topics. So if something is updated or something interesting is in the context of this topic, that you might have a button there, because actually it's all fairly the same, so you can read through these - different - yes, I'll say, posts, but there's nothing more that changes. Once you have read through everything, then you have read everythingt, then I actually just look to see if there's any news. So that might be something that would not be that uninteresting, I think.* (Interview 4, TP 16) |
